# Single and combined chronic toxicity of polystyrene nanoplastics (PSNP) and clothianidin on collembolans and enchytraeids

**DOI:** 10.1007/s11356-026-37540-2

**Published:** 2026-03-03

**Authors:** Felipe Ogliari Bandeira, Paulo Roger Lopes Alves, Tamires Rodrigues dos Reis, Dilmar Baretta, Carolina Riviera Duarte Maluche Baretta, William Gerson Matias

**Affiliations:** 1 Laboratory of Environmental Toxicology, Department of Sanitary and Environmental Engineering, Federal University of Santa Catarina (UFSC), Florianópolis, SC 88040-970 Brazil; 2 Laboratory of Soil Ecotoxicology, Universidade Federal da Fronteira Sul (UFFS), Chapecó, SC 89815-899 Brazil; 3 Department of Soil Science, Federal University of Lavras (UFLA), Lavras, MG 37200-900 Brazil; 4 Department of Animal Science, Center for Higher Education of the West, Santa Catarina State University (UDESC), Chapecó, SC 89815-630 Brazil; 5 Graduate Program in Environmental Sciences, Community University of Chapecó Region (Unochapecó), Chapecó, SC 89809-900 Brazil

**Keywords:** Additivity, Emerging contaminants, Mixture toxicity, Neonicotinoids, Plastic pollution, Soil mesofauna

## Abstract

This study assessed the chronic toxicity of polystyrene nanoplastics (PSNP) and clothianidin (CLO), individually and as a binary mixture, on enchytraeids *Enchytraeus crypticus* and collembolans *Folsomia candida*. The assays were performed according to ISO protocols, using a tropical soil (Entisol) as the test substrate. The mixture assays were performed using equitoxic concentration, standardized according to the preliminary EC_50_ values for each contaminant (1 Toxic Unit = reproductive EC_50_ average), and the toxicological interactions were assessed through the Abbott model. Individually, CLO was much more toxic than PSNP (LOEC-based) to both enchytraeids (CLO = 2.67 mg kg^−1^, PSNP = 128 mg kg^−1^) and collembolans (CLO = 0.02 mg kg^−1^, PSNP = 56.25 mg kg^−1^). Collembolans were more sensitive to both contaminants compared to enchytraeids, but the difference in sensitivity was higher for CLO (> 100 times) than for PSNP (2.3 times). The binary mixtures showed additive effects of PSNP + CLO for both species at all mixture concentrations. For collembolans, concentrations that caused no significant effects individually (18.75 mg PSNP kg^−1^ and 0.01 mg CLO kg^−1^) induced significant negative effects when in mixture, indicating that the co-occurrence of individually non-toxic concentrations might not be safe for collembolans.

**Supplementary Information:**

The online version contains supplementary material available at 10.1007/s11356-026-37540-2.

## Introduction

Contamination caused by plastics is considered a global environmental problem (Pilapitiya and Ratnayake 2024). Nanoplastics are currently one of the most studied classes of emerging contaminants (Moteallemi et al. 2024) and are usually classified as plastic particles with dimensions below 100 nm (nm) (Stapleton 2019). The occurrence and dynamics of nanoplastics in water bodies have been extensively investigated in recent years (Vicentini et al. 2019; Zhang et al. 2021; Delgado-Gallardo et al. 2021; Liu et al. 2024a, b; Shi et al. 2023; Choi et al. 2024; Bandeira et al. 2025), whereas their presence in soil ecosystems remains relatively understudied (Seo et al. 2024; Leal et al. 2025).

Nanoplastics may infiltrate agricultural soils via various routes, including atmospheric deposition (Wang et al. 2023), the degradation of plastic-based mulch films used in soil coverage applications (Ding et al. 2022), and the use of wastewater and biosolids, respectively, in the irrigation and organic fertilization of agricultural soils (Pérez-Reverón et al. 2022; Marchuk et al. 2023). Their presence in the soil can promote various negative effects on soil faunal organisms, including weight loss (Zhu et al. 2018), morphological damage on intestinal tissues (Tang et al. 2023), energy budget imbalance, and reduced egg formation and hatching (Huang et al. 2022) as well as gut microbiota alterations and oxidative damage (Barreto et al. 2023b).

In addition to the potential deleterious effects of nanoplastic particles on edaphic organisms, there is also substantial concern about the influence of these materials on the dynamics and toxicity of coexisting contaminants (Mendes et al. 2022; Barreto et al. 2023a). For instance, neonicotinoid-based insecticides are frequently found in agricultural environments (Schaafsma et al. 2015, 2016; Hua et al. 2023). These compounds are known to act as agonists of the insects’ nicotinic acetylcholine receptors (nAChRs), exerting great activity against the target pests with relatively low risk to mammals (Andrew and Samuels 2024).

Clothianidin (CLO) is one of the neonicotinoids widely employed in the prophylactic treatment of agricultural seeds in numerous countries (Alford and Krupke 2017; Lima et al. 2023). The recommended application doses of CLO for seed dressing often vary between 60 and 270 g active ingredient (a.i.) per 100 kg of seeds (Sumitomo Chemical 2024), which can ultimately result in soil concentrations ranging from 0.02 to 0.04 mg kg^−1^, depending on the sowing density and incorporation depth (Bandeira et al. 2021). Previous works have shown that these levels can be toxic to soil mesofauna organisms, negatively impacting collembolan growth (Alves et al. 2015) and reproduction (Bandeira et al. 2021, Graciani et al. 2023). In addition, detectable residues of CLO were already found ten months after the application (Botías et al. 2015), indicating that this compound can persist for long periods, accumulating in soil over successive applications (Xu et al. 2015) and coexisting with other pollutants in soil.

The dynamics of plastic fragments in combination with neonicotinoids in soil have been studied recently (Šunta et al. 2020; Xu et al. 2020; Hu et al. 2023; Zhou et al. 2024; García-Hernández et al. 2025). Although the toxicological effects on soil fauna remain relatively understudied, several authors have shown that micro- and nanoplastics can serve as vectors for transporting soil contaminants, including pesticides, and modify their original toxicity (Arikan et al. 2022; Wang et al. 2024; Baihetiyaer et al. 2023). For instance, Baihetiyaer et al. (2023) verified that 1% polylactic acid (PLA) biodegradable microplastics have not impaired *Eisenia fetida* reproduction when tested alone, but its mixture with the neonicotinoid imidacloprid (0.37 mg kg^−1^) amplified the deleterious effect toward the number of juveniles when compared to imidacloprid alone.

Other authors (Fu et al. 2023) have also found that the simultaneous exposure to imidacloprid (ranging from 0.1 to 1 mg kg soil^−1^) and 10 µm polyethylene microplastics (1% w/w) increased the epidermal damage induced on *E. fetida*, although no amplified negative effects of the mixtures were observed for acute toxicity (mortality). These studies used earthworms as the model organism for ecotoxicity tests, but mixture toxicity assessments with other taxonomic groups, such as collembolans and enchytraeids, are still needed to better understand the ecotoxicological impacts on organisms with different exposure pathways to soil contaminants. To date, no studies investigating the combined ecotoxicological effects of nanoplastics and clothianidin on collembolans and enchytraeids were found in the literature and we hypothesize that these mixtures may impact different soil fauna species in distinct ways.

The present study set out to evaluate the individual and combined ecotoxicological effects of polystyrene nanoplastics (PSNP) and CLO on two soil faunal species (collembolans *Folsomia candida* and enchytraeids *Enchytraeus crypticus*). Chronic toxicity assays were performed with the individual compounds and their mixtures according to the International Organization for Standardization (ISO) guidelines, and the ecotoxicological interaction between the mixtures was assessed using the Abbott model (Abbott 1925).

## Materials and methods

### Test soil and substances

The Brazilian natural tropical soil used in our experiments was taken from the topsoil layer (0–20 cm) of an area in Araranguá, Santa Catarina, Brazil, located in a region without prior pesticide use at the coordinates 29°00′S and 49°31′W. The sample was then defaunated (Bandeira et al. 2022), air-dried, and sieved at 2.0 mm. According to the classification framework established by the Brazilian Soil Classification System, the soil is a *Neossolo Quartzarênico órtico típico* (Santos et al. 2018) and corresponds to an Entisol in the Soil Taxonomy classification (Soil Survey Staff 2022). The water holding capacity (WHC) of Entisol was measured following Annex C of ISO 11268-2 (ISO 2012). Before conducting the assays, the moisture content of all treatments was adjusted to approximately 60% of the WHC of the Entisol.

Polystyrene nanoplastics (PSNP) were produced in the lab using the emulsion polymerization technique, following the procedure described in Vicentini et al. (2019). The polymerization process occurred at 70 °C, over 24 h, and the chemicals used were styrene monomer, Al_2_O_3_ nanoparticles, potassium persulfate, and sodium decyl sulfate (SDS). The synthesized PSNP particles are negatively charged, have a spherical shape, and have a mean particle size of 92.6 ± 4.4 nm (please see Bandeira et al. (2025) for additional details). A stock solution composed of 12 mg PSNP mL^−1^ was used to spike soil samples.

Clothianidin (CLO) was tested through the commercial formulation for seed treatment Inside FS, which contains 600 g CLO L^−1^. A stock solution of 6 mg CLO mL^−1^ was established and used in the soil contamination procedures.

The nominal PSNP and clothianidin concentrations tested ranged from 18.75–150 and 0.01–0.09 mg of active ingredient per kilogram of dry soil (mg kg⁻^1^) for collembolans and 64–512 and 0.67–5.34 mg kg^−1^ for enchytraeids, respectively (Table 1). The concentrations were chosen taking into account the results of the preliminary tests (Fig. S1), from which the individual EC_50_ values were obtained (Table S1). The concentration range was selected to ensure that a clear dose-response effect on organisms’ reproduction could be observed, which was necessary to quantify inhibition effects and to assess ecotoxicological interactions between the compounds. The mixture assays were performed using equitoxic concentrations of PSNP and CLO, which were normalized as Toxic Units (with 1 TU equivalent to the EC50), and the following range of TUs was evaluated for each compound: 0.25, 0.50, 0.75, 1, 1.5, and 2 TU.

**Table 1:** Concentrations adopted for the chronic toxicity assays with *Folsomia candida* and *Enchytraeus crypticus* exposed to individual polystyrene nanoplastic (PSNP) or clothianidin (CLO) and their mixtures (PSNP + CLO). TU = toxic unit (1 TU = EC_50_)

Species	Tested concentration		
	PSNP mg kg^−1^ (TU)	CLO mg kg^−1^ (TU)	PSNP + CLO mg kg^−1^ (TU)
*Folsomia candida*	18.7 (0.25)	0.01 (0.25)	18.7 + 0.01 (0.50)
	37.5 (0.50)	0.02 (0.50)	37.5 + 0.02 (1)
	56.2 (0.75)	0.03 (0.75)	56.2 + 0.03 (1.50)
	75 (1)	0.04 (1)	75 + 0.04 (2)
	112 (1.5)	0.07 (1.5)	112 + 0.07 (3)
	150 (2)	0.09 (2)	150 + 0.09 (4)
*Enchytraeus crypticus*	64 (0.25)	0.67 (0.25)	64 + 0.67 (0.50)
	128 (0.50)	1.33 (0.50)	128 + 1.33 (1)
	192 (0.75)	2 (0.75)	192 + 2 (1.50)
	256 (1)	2.67 (1)	256 + 2.67 (2)
	384 (1.5)	4 (1.5)	384 + 4 (3)
	512 (2)	5.34 (2)	512 + 5.34 (4)

### Chronic toxicity assays

Following ISO standards (ISO 2004; 2014), chronic toxicity tests involving collembolans and enchytraeids were carried out inside a temperature-regulated room set at 20 ± 2 °C under a 12-h light/dark cycle. For both species, the assays with the individual compounds and with the PSNP + CLO mixtures were run in parallel. Soil moisture and pH were quantified on the first and last day of each test (Table S2).

#### Collembola toxicity assays

Experiments involving collembolans utilized the species *F. candida* in accordance with the standardized protocol from ISO (ISO 2014). Six PSNP and CLO concentrations were tested, plus their binary mixtures (Table 1). A control group using only distilled water to moisten the soil was also set up. Ten randomly chosen collembolans were introduced into 200 mL snap-cap glass flasks filled with 30 g of moist soil, either treated or control. The age of the organisms was synchronized to guarantee that all the groups of organisms used in the tests were 10 to 12 days old. Ten replicates served as controls, while six were assigned to each treatment group. Collembolans were fed with a dose of *Saccharomyces cerevisiae* on the first day and at the midpoint of the test. A few drops of distilled water were used to adjust soil moisture every week. Following 28 days of exposure, the number of juveniles produced was recorded using the methodology described by Bandeira et al. (2020).

#### Enchytraeid toxicity assay

The chronic experiments employed enchytraeids of the species *E. crypticus*, as outlined by ISO (2004). Six PSNP and CLO concentrations were tested individually, plus their mixtures (Table 1). Ten individuals, about 1 cm long and with a visible clitellum, were placed in 200 mL glass flasks containing 30 g of wet soil (either contaminated or control). Oat flakes were provided as food on a weekly basis. The experiment included eight control replicates and five replicates for each treatment involving PSNP, CLO, or their combinations. New food was added, and soil moisture was adjusted with distilled water every 7 days. At the end of 21 days, 80% ethanol was introduced into each flask to fix both adults and offspring. Subsequently, they were stained with about five drops of a 1% Bengal rose xanthene dye solution in ethanol, and the juveniles produced were counted following the procedure described in Alves et al. (2015).

### Data analysis

#### Estimation of effect concentrations

Statistical analyses were performed using Statistica® software. Each dataset was individually examined for homoscedasticity via Bartlett’s test, followed by an assessment of normality with the Kolmogorov-Smirnov test. After confirming the analysis of variance (ANOVA) assumptions were fulfilled, the one-way ANOVA was run and Dunnett’s post hoc test was used to compare the mean number of juveniles found in contaminated soils with the respective control treatment, allowing establishment of the lowest tested concentration that is significantly different from control (LOEC) and the no observed effect concentration (NOEC). The concentrations that decreased reproduction by 50% (EC_50_ with its 95% confidence interval) were calculated by applying a logistic regression model (Environmental Canada 2007).

#### Assessment of the binary mixture interactions

The toxicological interactions between PSNP and CLO in the mixtures were assessed using the Abbott model (Abbott 1925). The mixture expected inhibition (*C*_exp_) was calculated using the toxic effects of the individual compounds on the species’ reproduction (PSNP and CLO separately), following Eq. 1.

1 $$C_{\mathrm{exp}}\;(\%)=A + B - (AB/100)$$

where *A* and *B* are the percent inhibition effects caused by PSNP and CLO as single compounds, respectively, on the number of produced juveniles (compared to the control). The observed inhibition (*C*_obs_) was also calculated as the percent inhibition induced by the mixtures (PSNP + CLO). Equation 2 was used to calculate the ratio of inhibition (RI).

2 $$\mathrm{RI}=C_{\mathrm{obs}}/C_{\mathrm{exp}}$$

where RI values = 1 indicate an additive effect, RI > 1 indicate potential synergism, and RI < 1 indicate potential antagonism. To check for significant deviations of the additive model, the *C*_obs_ was statistically compared with the *C*_exp_ for each combination of concentrations tested using Duncan’s test (*p* < 0.05). Interactions were only considered significant for antagonism or synergism when the *C*_obs_ significantly differed from *C*_exp_ (*p* < 0.05), whereas *p* > 0.05 was assumed as additive effects.

## Results

**Fig. 1:**
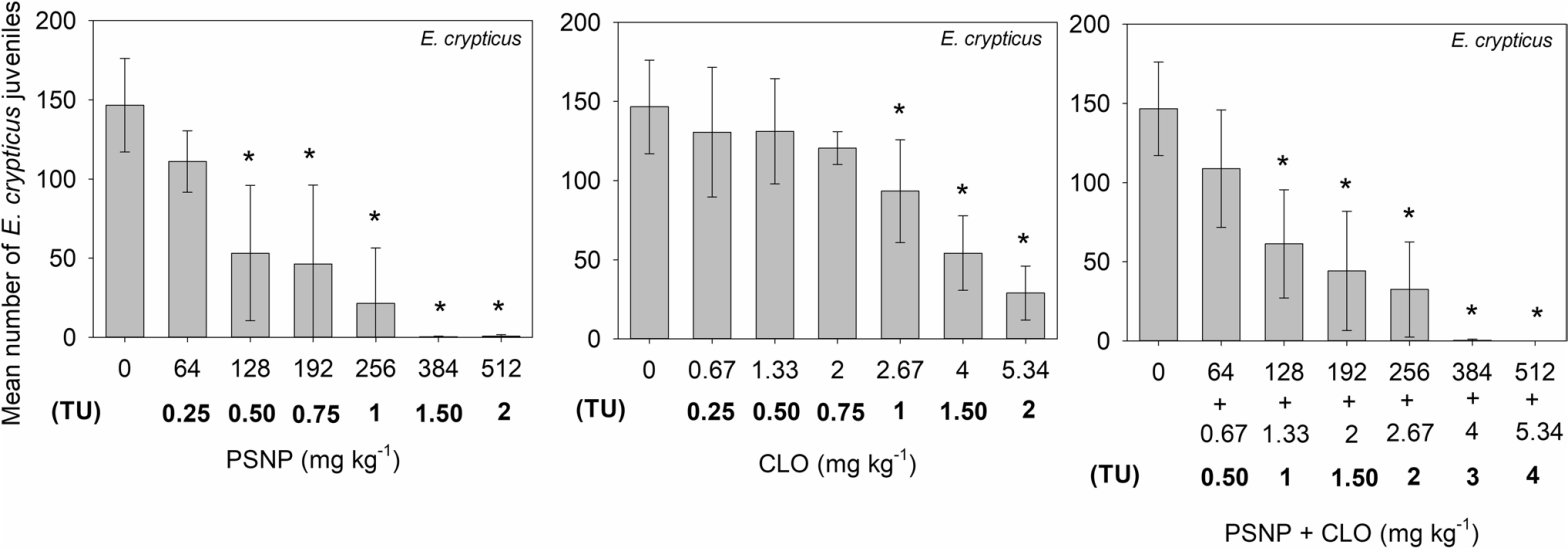
Mean number of *Enchytraeus crypticus* juveniles (± standard deviation, *n* = 5) found in Entisol contaminated with increasing concentrations of polystyrene nanoplastic (PSNP), clothianidin (CLO) and their mixtures (PSNP + CLO). TU = toxic unit (1 TU = EC_50_)

**Fig. 2:**
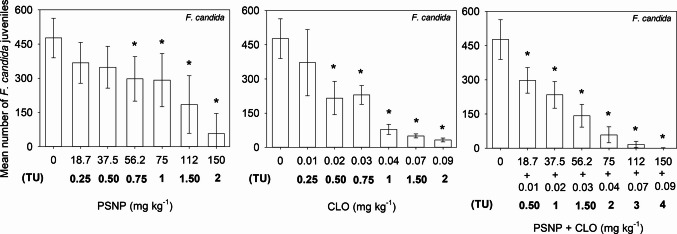
Mean number of *Folsomia candida* juveniles (± standard deviation, *n* = 6) found in Entisol contaminated with increasing concentrations of polystyrene nanoplastic (PSNP), clothianidin (CLO) and their mixtures (PSNP + CLO). TU = toxic unit (1 TU = EC_50_)

When tested individually, PSNP significantly reduced enchytraeid and collembolan reproduction from 128 mg kg^−1^ to 56.2 mg kg^−1^, respectively. CLO was comparatively more toxic than PSNP for both species, showing notable declines (*p* < 0.05) in juvenile production starting at 2.67 mg kg^−1^ for enchytraeids (Fig. 1) and 0.02 mg kg^−1^ for collembolans (Fig. 2).

Mixture toxicity assays showed that the observed inhibition (*C*_obs_) was similar to the expected inhibition (*C*_exp_, predicted by the Abbott model) for all the tested combinations of PSNP and CLO, revealing that the interaction between PSNP and CLO for both species is predominantly additive (Table 2). For *E. crypticus*, significant negative effects (*p* < 0.05) were observed from 128 mg PSNP kg^−1^ + 1.33 mg CLO kg^−1^ (Fig. 1). A significant statistical decrease (*p* < 0.05) in the number of juveniles generated by collembolans was observed from the mixture of 18.7 mg PSNP kg^−1^ + 0.01 mg CLO kg^−1^, which did not cause significant decreases (*p* > 0.05) when tested as single compounds (Fig. 2).

**Table 2:** Percentage of inhibitory effect induced by polystyrene nanoplastic (PSNP), clothianidin (CLO) and their mixtures (PSNP + CLO) on *Enchytraeus crypticus* and *Folsomia candida* reproduction

Species	PSNP (mg kg^−1^) [TU ^a^]	A^b^	CLO (mg kg^−1^) [TU ^a^]	B^c^	Expected inhibition (C_exp_) (%)^d^	PSNP + CLO (mg kg^−1^) [TU^a^]	Observed inhibition (C_obs_) (%)^e^	RI^f^	Interaction nature	*p-*value
*E. crypticus*	64 [0.25]	24.2 ± 13.3	0.67 [0.25]	10.9 ± 27.9	31.8 ± 19.9	64 + 0.67 [0.50]	25.7 ± 25.2	0.81	Additive effect	0.604 g
	128 [0.50]	63.7 ± 29.2	1.34 [0.50]	10.5 ± 22.7	68.9 ± 24.4	128 + 1.34 [1.00]	58.1 ± 23.3	0.84	Additive effect	0.382 g
	192 [0.75]	68.3 ± 33.9	2.00 [0.75]	17.7 ± 7.1	73.9 ± 28.2	192 + 2.00 [1.50]	69.8 ± 25.8	0.94	Additive effect	0.770 g
	256 [1.00]	85.4 ± 23.9	2.67 [1.00]	36.3 ± 22.2	92.0 ± 12.6	256 + 2.67 [2.00]	77.8 ± 20.5	0.86	Additive effect	0.118 g
	384 [1.50]	99.8 ± 0.3	4.01 [1.50]	63.0 ± 16.1	99.9 ± 0.2	384 + 4.01 [3.00]	99.7 ± 0.5	1.00	Additive effect	0.391 g
	512 [2.00]	99.6 ± 0.7	5.34 [2.00]	80.2 ± 11.6	99.9 ± 0.1	512 + 5.34 [4.00]	100 ± 0.0	1.00	Additive effect	0.079 g
*F. candida*	18.7 [0.25]	22.9 ± 18.7	0.01 [0.25]	22.1 ± 30.5	41.3 ± 24.6	18.7 + 0.01 [0.50]	37.5 ± 11.7	0.91	Additive effect	0.743 g
	37.5 [0.50]	26.9 ± 19.2	0.02 [0.50]	54.6 ± 15.3	67.9 ± 10.9	37.5 + 0.02 [1.00]	53.8 ± 10.0	0.79	Additive effect	0.061 g
	56.2 [0.75]	37.6 ± 20.4	0.03 [0.75]	51.8 ± 8.6	69.4 ± 13.6	56.2 + 0.03 [1.50]	70.1 ± 10.5	1.01	Additive effect	0.919 g
	75.0 [1.00]	38.7 ± 24.5	0.04 [1.00]	83.5 ± 4.7	89.5 ± 5.4	75.0 + 0.04 [2.00]	87.8 ± 7.3	0.98	Additive effect	0.652 g
	112 [1.50]	61.2 ± 26.5	0.07 [1.50]	89.5 ± 1.9	95.9 ± 2.7	112 + 0.07 [3.00]	96.6 ± 3.0	1.01	Additive effect	0.668 g
	150 [2.00]	88.1 ± 18.7	0.09 [2.00]	93.3 ± 1.8	99.1 ± 1.4	150 + 0.09 [4.00]	99.9 ± 0.2	1.01	Additive effect	0.230 g

^a^*TU* toxic unit. 1 TU = EC_50_

^b^*A* inhibitory effect induced by PSNP

^c^*B* inhibitory effect induced by CLO

^d^*C*_*exp*_ expected inhibition calculated by Abbott’s formula: *C*_exp_ (%) = A + B − (AB/100)

^e^*C*_*obs*_ percent inhibition induced by the mixtures (PSNP + CLO)

^f^*RI* ratio of inhibition. *RI *observed inhibition/expected inhibition

^g^*p* > 0.05 (Duncan’s test) for the comparison of *C*_exp_ with *C*_obs_, indicating an additive effect

## Discussion

Individually, PSNP caused low toxicity to both species, with significant effects (*p* < 0.05) being observed only above 50 and 100 mg kg^−1^ for collembolans and enchytraeids, respectively. These findings are in line with the outcomes reported in other papers. For instance, Barreto et al. (2020) observed negative effects of polystyrene nanoplastics (mean diameter 49 nm) on *E. crypticus* starting at 1200 mg kg^−1^. In another study with collembolans, Barreto et al. (2023a) observed no negative effects of polystyrene nanoplastics (45 nm) on *F. candida* survival and reproduction up to 600 mg kg^−1^. The lower toxicity found by Barreto et al. (2020, 2023a) could be related to the use of the LUFA 2.2 soil, which has a higher amount of organic matter (4.5%, Van Hall et al. 2025) compared to our Entisol (2.2%, Bandeira et al. 2020).

The mechanisms responsible for the harmful effects of plastic nanoparticles on soil faunal organisms still need to be better clarified, but they are generally linked with tissue abrasion, intestinal channel blockage and microbiota dysregulation (Zhu et al. 2017, 2018), alongside the triggering of oxidative stress responses (Kim et al. 2018).

CLO was more toxic than PSNP, with significant reductions (*p* < 0.05) in the enchytraeids and collembolans reproduction being observed from 2.67 mg kg^−1^ (Fig. 1) and 0.02 mg kg^−1^ (Fig. 2). LOEC values also revealed a higher sensitivity to CLO in collembolans compared to enchytraeids, which is in line with our previous work using an artificial tropical soil (Bandeira et al. 2021), where *E. crypticus* was only negatively affected in a much higher concentration (40 mg kg^−1^) than collembolans (0.08–0.18 mg kg^−1^). Similar to our results, previous studies also reported high toxicity of CLO to collembolans when exposure occurred in sandy soils. Graciani et al. (2023) reported a clothianidin EC_50_ of 0.030 (0.022–0.039) mg kg^−1^ for *F. candida* in the same Entisol used in our study, and Ritchie et al. (2019) found a clothianidin EC_50_ of 0.069 (0.039–0.12) mg kg^−1^ for *F. candida* in a natural sandy loam soil with 2.5 ± 0.6% organic matter.

The results of the mixture assays (Table 2) indicate that the toxic effects of the mixture between PSNP and CLO correspond to the sum of the individual effects observed for each molecule, since the interactions were predominantly additive at all concentrations tested. Mixture additive effects were observed for both species, indicating that our hypothesis of distinct mixture interactions for the species tested was rejected. A possible explanation for these results is related to the different mechanisms of action and adsorption sites of PSNP and CLO. While nanoplastics act mainly by interrupting the digestive system and resulting in energy assimilation losses (Huang et al. 2022) and generating reactive oxygen species due to internalization in different cells (Nogueira et al. 2021), CLO acts specifically on nicotinic acetylcholine receptors, causing interruption in the transmission of nerve impulses (Andrew and Samuels 2024). Therefore, it is likely that the mechanism of action of one compound did not notably interfere with the action of the other, which may help account for the additive effects observed in the mixture.

For *E. crypticus*, the mixture only caused toxicity at concentrations that had already caused a significant reduction in reproduction individually. However, for *F. candida*, the combination of individually non-toxic individual (18.7 mg of PSNP kg^−1^ + 0.01 mg of CLO kg^−1^) caused a significant toxic effect, indicating that the coexistence of these contaminants, even when at theoretically safe individual levels, may represent an ecotoxicological threat for these soil microarthropods. These results also warn of the indirect effect of plastic particles on the toxicity of other contaminants, potentially reflecting facilitated uptake of CLO via adsorption onto the PSNP surface, which may increase its bioavailability to soil organisms. Differences in nanoplastic surface functionalization and charge may further modulate these interactions with neonicotinoids, influencing adsorption behavior, bioavailability, and toxicity (Hüffer and Hofmann 2016; Barreto et al. 2023c). In line with our findings, Baihetiyaer et al. (2023) verified that 1% polylactic acid (PLA) biodegradable microplastics, when tested individually, caused no effects on *E. fetida* reproduction, but amplified the toxic effects of imidacloprid (0.37 mg kg^−1^) when in mixture.

The number of studies quantifying nanoplastics concentration in soil environments is scarce. For instance, nanoplastic (20–150 nm) concentrations of 23.7 ± 1.8 mg kg^−1^ were detected in household waste-contaminated agricultural soils in France (Wahl et al. 2021; Liu et al. 2024a, b). Microplastics, on the other hand, are more easily quantified in soil. Büks and Kaupenjohann (2020) reviewed 23 studies that have measured microplastic concentration throughout 223 distinct sampling sites across the globe and found that, on average, microplastic levels in agricultural soils amount to approximately 1.7 mg kg^−1^. Therefore, the range of PSNP concentrations that cause negative effects in our experiments (Figs. 1 and 2) likely exceeds the typical concentration range observed in agricultural soils.

On the other hand, residual levels of CLO after planting treated seeds were detected in agricultural soils within the range of concentrations adopted in our experiments. As an example, Jones et al. (2014) identified CLO levels between 0.00002 and 0.0136 mg kg^−1^ in cultivated soils across England. Similarly, Woodward et al. (2021) detected clothianidin residues in soil at concentrations up to 0.029 mg kg^−1^ within a lettuce plantation in the USA where seeds treated with clothianidin had been sown. Although the clothianidin concentrations detected by the authors in agricultural fields are potentially safe for *E. crypticus*, they can be toxic to *F. candida*, as we observed significant reductions (*p* < 0.05) in collembolan reproduction starting at 0.02 mg kg^−1^ when clothianidin was tested individually and at 0.01 mg kg^−1^ when clothianidin was in a mixture with PSNP (Fig. 2).

Research exploring how nanoplastics affect neonicotinoid toxicity remains at an early stage. Nonetheless, earlier findings have shown that microplastics may amplify the ecotoxicity of pesticides, as they can adsorb pesticide molecules on their surface and facilitate the internalization of the exposed organisms (Cheng et al. 2020; Sun et al. 2020). For example, microplastics at a concentration of 10 mg kg⁻^1^ (with sizes between 40 and 50 µm) increased dufulin bioaccumulation in the earthworm species *E. fetida* (Sun et al. 2020). Similarly, increased pesticide bioaccumulation by earthworms in the presence of microplastics was reported for atrazine (Cheng et al. 2020; Song et al. 2023), phenanthrene (Xu et al. 2021) and 2,4-D (Boughattas et al. 2022). As evidenced by Boughattas et al. (2022), microplastic particles can modify the behavior and toxic potential of pesticides at environmentally relevant (100 µg kg^−1^) concentrations. In this study, the PSNP concentrations tested were likely much higher than the currently predicted environmental levels. This approach was adopted to ensure measurable inhibitory effects, allowing the assessment of binary mixture interactions using the Abbott model. As studies addressing PSNP–clothianidin mixture toxicity in soil invertebrates remain scarce, this work provides one of the first ecotoxicological evaluations of PSNP–CLO mixtures in collembolans and enchytraeids. However, for future ecotoxicological evaluations, it is recommended to investigate the influence of environmental concentrations (< 1 mg kg^−1^) of nanoplastics on the toxicity of neonicotinoids, since concentrations much lower than those tested in this study may interfere with the toxic level of neonicotinoids.

## Conclusion

Based on the individual toxic effects verified in this study, we can conclude that CLO is more harmful to both collembolans and enchytraeids than PSNP. Also, collembolans should be prioritized for mixture toxicity evaluations of nanoplastics and neonicotinoids, due to their increased sensitivity when compared to enchytraeids. We also conclude that the combined effects of PSNP and CLO on the studied species’ reproduction can be predicted by the additive model, as no deviation from the model was identified in any of the concentrations tested, thus rejecting our hypothesis of distinct mixture interactions for the different species. However, for collembolans, the combination of individually non-toxic concentrations of PSNP and CLO resulted in significant negative effects on *F. candida* reproduction. Our findings reinforce the importance of ecotoxicological assessment of mixtures of plastic particles and neonicotinoids and contribute to the regulation of emerging contaminants.

## Supplementary Information

Below is the link to the electronic supplementary material.

ESM 1 (DOCX 207 KB)

## Data Availability

The raw data supporting the findings of this study is available and should be requested by email.

## References

[CR1] Abbott WS (1925) A method of computing the effectiveness of an insecticide. J Econ Entomol 18:265–267

[CR2] Alford A, Krupke CH (2017) Translocation of the neonicotinoid seed treatment clothianidin in maize. PLoS ONE 12(3):e0173836. 10.1371/journal.pone.0173836 28282441 10.1371/journal.pone.0173836 PMC5345846

[CR3] Alves PRL, Natal-Da-Luz T, Sousa JP, Cardoso EJBN (2015) Ecotoxicological characterization of sugarcane vinasses when applied to tropical soils. Sci Total Environ 526:222–232. 10.1016/j.scitotenv.2015.03.150 25933292 10.1016/j.scitotenv.2015.03.150

[CR4] Andrew D, Samuels S (2024) Cholinergic neurotransmission and toxicity neonicotinoids and spinosad. In Royal Society of Chemistry eBooks (pp. 49–90). 10.1039/9781839165795-00049

[CR5] Arikan B, Ozfidan-Konakci C, Yildiztugay E, Turan M, Cavusoglu H (2022) Polystyrene nanoplastic contamination mixed with polycyclic aromatic hydrocarbons: alleviation on gas exchange, water management, chlorophyll fluorescence and antioxidant capacity in wheat. Environ Pollut 311:119851. 10.1016/j.envpol.2022.119851 35987286 10.1016/j.envpol.2022.119851

[CR6] Baihetiyaer B, Jiang N, Li X, Song J, Wang J, Fan X, Zuo Y, Yin X (2023) Exploring the toxicity of biodegradable microplastics and imidacloprid to earthworms (*Eisenia fetida*) from morphological and gut microbial perspectives. Environ Pollut 337:122547. 10.1016/j.envpol.2023.122547 37709123 10.1016/j.envpol.2023.122547

[CR7] Bandeira FO, Alves PRL, Hennig TB, Toniolo T, Natal-da-Luz T, Baretta D (2020) Effect of temperature on the toxicity of imidacloprid to *Eisenia andrei* and *Folsomia candida* in tropical soils. Environ Pollut 267:115565. 10.1016/j.envpol.2020.115565 33254719 10.1016/j.envpol.2020.115565

[CR8] Bandeira FO, Alves PRL, Hennig TB, Brancalione J, Nogueira DJ, Matias WG (2021) Chronic effects of clothianidin to non-target soil invertebrates: Ecological risk assessment using the species sensitivity distribution (SSD) approach. J Hazard Mater 419:126491. 10.1016/j.jhazmat.2021.126491 10.1016/j.jhazmat.2021.126491 34323739

[CR9] Bandeira FO, Alves PRL, Hennig TB, Vaz VP, Vicentini DS, Juneau P, Dewez D, Matias WG (2025) Individual and combined toxicity of polystyrene nanoplastics and clothianidin toward *Daphnia magna*, *Lemna minor*, *Chlamydomonas reinhardtii*, and *Microcystis aeruginosa*. Environ Toxicol Chem 44:470–483. 10.1093/etojnl/vgae029 39919234 10.1093/etojnl/vgae029

[CR10] Bandeira FO, Lodi MR, Graciani TS, Oroski S, Mattias JL, Cardoso EJBN, Alves PRL (2022) The use of sewage sludge as remediation for imidacloprid toxicity in soils. Environ Sci Pollut Res 30(8):20159–20167. 10.1007/s11356-022-23584-7 10.1007/s11356-022-23584-7 36251199

[CR11] Barreto A, Santos J, Amorim MJB, Maria VL (2020) How Can Nanoplastics Affect the Survival Reproduction and Behaviour of the Soil Model Enchytraeus crypticus? Appl Sci 10(21):7674. 10.3390/app10217674

[CR12] Barreto A, Santos J, Almeida L, Tavares V, Pinto E, Celeiro M, Garcia-Jares C, Maria VL (2023a) First approach to assess the effects of nanoplastics on the soil species *Folsomia candida*: a mixture design with bisphenol A and diphenhydramine. NanoImpact 29:100450. 10.1016/j.impact.2023.100450 36610661 10.1016/j.impact.2023.100450

[CR13] Barreto A, Santos J, Andrade G, Santos M, Maria VL (2023b) New insights into nanoplastics ecotoxicology: effects of long-term polystyrene nanoparticles exposure on *Folsomia candida*. Toxics 11(10):876. 10.3390/toxics11100876 37888726 10.3390/toxics11100876 PMC10610651

[CR14] Barreto M, Lopes I, Oliveira M (2023c) Micro(nano)plastics: a review on their interactions with pharmaceuticals and pesticides. Trends Anal Chem 169:117307. 10.1016/j.trac.2023.117307

[CR15] Botías C, David A, Horwood J, Abdul-Sada A, Nicholls E, Hill E, Goulson D (2015) Neonicotinoid residues in wildflowers, a potential route of chronic exposure for bees. Environ Sci Technol 49:12731–12740. 10.1021/acs.est.5b03459 26439915 10.1021/acs.est.5b03459

[CR16] Boughattas I, Zitouni N, Hattab S, Mkhinini M, Missawi O, Helaoui S, Mokni M, Bousserrhine N, Banni M (2022) Interactive effects of environmental microplastics and 2,4-dichlorophenoxyacetic acid (2,4-D) on the earthworm *Eisenia andrei*. J Hazard Mater 424:127578. 10.1016/j.jhazmat.2021.127578 34736209 10.1016/j.jhazmat.2021.127578

[CR17] Büks F, Kaupenjohann M (2020) Global concentrations of microplastics in soils – a review. SOIL 6(2):649–662. 10.5194/soil-6-649-2020

[CR18] Cheng Y, Zhu L, Song W, Jiang C, Li B, Du Z, Wang J, Wang J, Li D, Zhang K (2020) Combined effects of mulch film-derived microplastics and atrazine on oxidative stress and gene expression in earthworm (*Eisenia fetida*). Sci Total Environ 746:141280. 10.1016/j.scitotenv.2020.141280 32745867 10.1016/j.scitotenv.2020.141280

[CR19] Choi S, Lee S, Kim M, Yu E, Ryu Y (2024) Challenges and recent analytical advances in micro/nanoplastic detection. Anal Chem 96(22):8846–8854. 10.1021/acs.analchem.3c05948 38758170 10.1021/acs.analchem.3c05948

[CR20] Delgado-Gallardo J, Sullivan GL, Esteban P, Wang Z, Arar O, Li Z, Watson TM, Sarp S (2021) From sampling to analysis: a critical review of techniques used in the detection of micro- and nanoplastics in aquatic environments. ACS ES&T Water 1(4):748–764. 10.1021/acsestwater.0c00228

[CR21] Ding F, Jones DL, Chadwick DR, Kim PJ, Jiang R, Flury M (2022) Environmental impacts of agricultural plastic film mulch: fate, consequences, and solutions. Sci Total Environ 836:155668. 10.1016/j.scitotenv.2022.155668

[CR22] Environmental Canada (2007) Guidance document on statistical methods for environmental toxicity test. Environmental Protection Series, EPS 1/RM/46, 2005 with 2007 Updates. Environmental Canada, Ottawa.

[CR23] Fu H, Zhu L, Mao L, Zhang L, Zhang Y, Chang Y, Liu X, Jiang H (2023) Combined ecotoxicological effects of different-sized polyethylene microplastics and imidacloprid on the earthworms (*Eisenia fetida*). Sci Total Environ 870:161795. 10.1016/j.scitotenv.2023.161795 36708821 10.1016/j.scitotenv.2023.161795

[CR24] García-Hernández E, Torres FJ, Cortés-Arriagada D, Nochebuena J (2025) Understanding the co-adsorption mechanism between nanoplastics and neonicotinoid insecticides from an atomistic perspective. J Mol Model 31(5):140. 10.1007/s00894-025-06364-1 40232319 10.1007/s00894-025-06364-1

[CR25] Graciani TS, Bandeira FO, Cardoso EJBN, Alves PRL (2023) Influence of temperature and soil moisture on the toxic potential of clothianidin to collembolan Folsomia candida in a tropical field soil. Ecotoxicology 32(1):82–92. 10.1007/s10646-023-02621-2 10.1007/s10646-023-02621-2 36648631

[CR26] Hu M, Huang L, Wang Y, Tan H, Yu X (2023) Insight into the effect of microplastics on the adsorption and degradation behavior of thiamethoxam in agricultural soils. Chemosphere 337:139262. 10.1016/j.chemosphere.2023.139262 37339706 10.1016/j.chemosphere.2023.139262

[CR27] Hua L, Zhao D, Wang H, Wei T (2023) Residues and bioavailability of neonicotinoid pesticide in Shaanxi agricultural soil. Water Air Soil Pollut 234:129. 10.1007/s11270-023-06159-1

[CR28] Huang C-W, Yen P-L, Kuo Y-H, Chang C-H, Liao VH-C (2022) Nanoplastic exposure in soil compromises the energy budget of the soil nematode C. elegans and decreases reproductive fitness. Environ Pollut 312:120071. 10.1016/j.envpol.2022.120071 10.1016/j.envpol.2022.120071 36055456

[CR29] Hüffer T, Hofmann T (2016) Sorption of non-polar organic compounds by micro-sized plastic particles in aqueous solution. Environ Pollut 214:194–201. 10.1016/j.envpol.2016.04.018 27086075 10.1016/j.envpol.2016.04.018

[CR30] ISO (2004) International Standardization Organization – 16387. Soil quality: effects of pollutants on Enchytraeidae (*Enchytraeus* sp.)—determination of effects on reproduction and survival. Genève, Switzerland.

[CR31] ISO (2012) International Standardization Organization - 11268–2. Soil quality – effects of pollutants on earthworms - part 2: determination of effects on reproduction of *Eisenia fetida*/*Eisenia andrei*. Genève, Switzerland.

[CR32] ISO (2014) International Standardization Organization - 11267. Soil quality – inhibition of reproduction of Collembola (*Folsomia candida*) by soil contaminants. Genève, Switzerland.

[CR33] Jones A, Harrington P, Turnbull G (2014) Neonicotinoid concentrations in arable soils after seed treatment applications in preceding years. Pest Manag Sci 70(12):1780–1784. 10.1002/ps.3836 24888990 10.1002/ps.3836

[CR34] Kim HM, Lee D, Long NP, Kwon SW, Park JH (2018) Uptake of nanopolystyrene particles induces distinct metabolic profiles and toxic effects in *Caenorhabditis elegans*. Environ Pollut 246:578–586. 10.1016/j.envpol.2018.12.043 30597390 10.1016/j.envpol.2018.12.043

[CR35] Leal TW, Tochetto G, De Medeiros Lima SV, De Oliveira PV, Schossler HJ, De Oliveira CRS, Da Silva Júnior AH (2025) Nanoplastics and microplastics in agricultural systems: effects on plants and implications for human consumption. Microplastics 4(2):16. 10.3390/microplastics4020016

[CR36] Lima AF, Aguirre NM, Carvalho GA, Grunseich JM, Helms AM, Peñaflor MFGV (2023) Effects of neonicotinoid seed treatment on maize anti-herbivore defenses vary across plant genotypes. J Pest Sci 97(1):199–212. 10.1007/s10340-023-01641-5

[CR37] Liu H, Yang J, Jiang Y, Sun R, Zhou P, Li J (2024a) Behavior of microplastics and nanoplastics in farmland soil environment and mechanisms of interaction with plants. Pol J Environ Stud 33(3):2499–2513. 10.15244/pjoes/175021

[CR38] Liu S, Chen Q, Ding H, Song Y, Pan Q, Deng H, Zeng EY (2024b) Differences of microplastics and nanoplastics in urban waters: environmental behaviors, hazards, and removal. Water Res 260:121895. 10.1016/j.watres.2024.121895 38875856 10.1016/j.watres.2024.121895

[CR39] Marchuk S, Tait S, Sinha P, Harris P, Antille DL, McCabe BK (2023) Biosolids-derived fertilisers: a review of challenges and opportunities. Sci Total Environ 875:162555. 10.1016/j.scitotenv.2023.162555 36889394 10.1016/j.scitotenv.2023.162555

[CR40] Mendes LA, Barreto A, Santos J, Amorim MJB, Maria VL (2022) Co-exposure of nanopolystyrene and other environmental contaminants—their toxic effects on the survival and reproduction of *Enchytraeus crypticus*. Toxics 10(4):193. 10.3390/toxics10040193 35448454 10.3390/toxics10040193 PMC9032828

[CR41] Moteallemi A, Dehghani MH, Momeniha F, Azizi S (2024) Nanoplastics as emerging contaminants: a systematic review of analytical processes, removal strategies from water environments, challenges and perspective. Microchem J 207:111884. 10.1016/j.microc.2024.111884

[CR42] Nogueira DJ, De Oliveira Da Silva AC, Da Silva MLN, Vicentini DS, Matias WG (2021) Individual and combined multigenerational effects induced by polystyrene nanoplastic and glyphosate in *Daphnia magna* (Strauss, 1820). Sci Total Environ 811:151360. 10.1016/j.scitotenv.2021.151360 34774938 10.1016/j.scitotenv.2021.151360

[CR43] Pérez-Reverón R, González-Sálamo J, Hernández-Sánchez C, González-Pleiter M, Hernández-Borges J, Díaz-Peña FJ (2022) Recycled wastewater as a potential source of microplastics in irrigated soils from an arid-insular territory (Fuerteventura, Spain). Sci Total Environ 817:152830. 10.1016/j.scitotenv.2021.152830 35016926 10.1016/j.scitotenv.2021.152830

[CR44] Pilapitiya PNT, Ratnayake AS (2024) The world of plastic waste: a review. Cleaner Materials 11:100220. 10.1016/j.clema.2024.100220

[CR45] Ritchie EE, Maisonneuve F, Scroggins RP, Princz JI (2019) Lethal and sublethal toxicity of thiamethoxam and clothianidin commercial formulations to soil invertebrates in a natural soil. Environ Toxicol Chem 38(10):2111–2120. 10.1002/etc.4521 31211447 10.1002/etc.4521

[CR46] Santos HG. Jacomine PKT, Anjos LHC dos, Oliveira VA de, Lumbreras JF, Coelho MR, Almeida JA de, Araujo Filho JC de, Oliveira JB de, Cunha TJF (2018) Sistema Brasileiro de Classificação de Solos. 5. ed. Brasília, DF: Embrapa, 531 p. ISBN 978–85–7035–817–2

[CR47] Schaafsma A, Limay-Rios V, Baute T, Smith J, Xue Y (2015) Neonicotinoid insecticide residues in surface water and soil associated with commercial maize (corn) fields in Southwestern Ontario. PLoS ONE 10(2):e0118139. 10.1371/journal.pone.0118139 25710560 10.1371/journal.pone.0118139 PMC4339550

[CR48] Schaafsma A, Limay-Rios V, Xue Y, Smith J, Baute T (2016) Field-scale examination of neonicotinoid insecticide persistence in soil as a result of seed treatment use in commercial maize (corn) fields in Southwestern Ontario. Environ Toxicol Chem 35:295–302. 10.1002/etc.3231 26332416 10.1002/etc.3231

[CR49] Seo Y, Zhou Z, Lai Y, Chen G, Pembleton K, Wang S, He J, Song P (2024) Micro- and nanoplastics in agricultural soils: assessing impacts and navigating mitigation. Sci Total Environ 931:172951. 10.1016/j.scitotenv.2024.172951 38703838 10.1016/j.scitotenv.2024.172951

[CR50] Shi C, Liu Z, Yu B, Zhang Y, Yang H, Han Y, Wang B, Liu Z, Zhang H (2023) Emergence of nanoplastics in the aquatic environment and possible impacts on aquatic organisms. Sci Total Environ 906:167404. 10.1016/j.scitotenv.2023.167404 37769717 10.1016/j.scitotenv.2023.167404

[CR51] Soil Survey Staff (2022) Keys to soil taxonomy, 13th edition. USDA Natural Resources Conservation Service.

[CR52] Song W, Du Y, Li D, Xiao Z, Li B, Wei J, Huang X, Zheng C, Wang J, Wang J, Zhu L (2023) Polyethylene mulch film-derived microplastics enhance the bioaccumulation of atrazine in two earthworm species (*Eisenia fetida* and *Metaphire guillelmi*) via carrier effects. J Hazard Mater 455:131603. 10.1016/j.jhazmat.2023.131603 37182465 10.1016/j.jhazmat.2023.131603

[CR53] Stapleton P (2019) Toxicological considerations of nano-sized plastics. AIMS Environ Sci 6(5):367–378. 10.3934/environsci.2019.5.367 31745497 10.3934/environsci.2019.5.367 PMC6863350

[CR54] Sumitomo Chemical (2024) INSIDE FS: Inseticida sistêmico para tratamento de sementes (in Portuguese). https://www.adapar.pr.gov.br/sites/adapar/arquivos_restritos/files/documento/2024-10/inside.pdf

[CR55] Sun W, Meng Z, Li R, Zhang R, Jia M, Yan S, Tian S, Zhou Z, Zhu W (2020) Joint effects of microplastic and dufulin on bioaccumulation, oxidative stress and metabolic profile of the earthworm (*Eisenia fetida*). Chemosphere 263:128171. 10.1016/j.chemosphere.2020.128171 33297140 10.1016/j.chemosphere.2020.128171

[CR56] Šunta U, Prosenc F, Trebše P, Bulc TG, Kralj MB (2020) Adsorption of acetamiprid, chlorantraniliprole and flubendiamide on different type of microplastics present in alluvial soil. Chemosphere 261:127762. 10.1016/j.chemosphere.2020.127762 32738715 10.1016/j.chemosphere.2020.127762

[CR57] Tang R, Zhu D, Luo Y, He D, Zhang H, El-Naggar A, Palansooriya KN, Chen K, Yan Y, Lu X, Ying M, Sun T, Cao Y, Diao Z, Zhang Y, Lian Y, Chang SX, Cai Y (2023) Nanoplastics induce molecular toxicity in earthworm: integrated multi-omics, morphological, and intestinal microorganism analyses. J Hazard Mater 442:130034. 10.1016/j.jhazmat.2022.130034 36206716 10.1016/j.jhazmat.2022.130034

[CR58] Van Hall BG, Sweeney CJ, Bottoms M, Van Gestel CAM (2025) The influence of soil organic matter content on the toxicity of pesticides to the springtail *Folsomia candida*. Environ Toxicol Chem 44:524–533. 10.1093/etojnl/vgae048 39919230 10.1093/etojnl/vgae048 PMC11816275

[CR59] Vicentini DS, Nogueira DJ, Melegari SP, Arl M, Köerich JS, Cruz L, Justino NM, Oscar BV, Puerari RC, Da Silva MLN, Simioni C, Ouriques LC, Matias MS, De Castilhos Junior AB, Matias WG (2019) Toxicological evaluation and quantification of ingested metal‐core nanoplastic by *Daphnia magna* through fluorescence and inductively coupled plasma‐mass spectrometric methods. Environ Toxicol Chem 38(10):2101–2110. 10.1002/etc.4528 31233230 10.1002/etc.4528

[CR60] Wahl A, Juge CL, Davranche M, Hadri HE, Grassl B, Reynaud S, Gigault J (2021) Nanoplastic occurrence in a soil amended with plastic debris. Chemosphere 262:127784. 10.1016/j.chemosphere.2020.127784 32777612 10.1016/j.chemosphere.2020.127784

[CR61] Wang K, Li C, Li H, Liu Q, Khan K, Li F, Chen W, Xu L (2024) Interactions of traditional and biodegradable microplastics with neonicotinoid pesticides. Sci Total Environ 947:174512. 10.1016/j.scitotenv.2024.174512 38972406 10.1016/j.scitotenv.2024.174512

[CR62] Wang Y, Xiang L, Amelung W, Elsner M, Gan J, Kueppers S, Christian L, Jiang X, Adu-Gyamfi J, Heng L, Ok YS, Ivleva NP, Luo Y, Barceló D, Schäffer A, Wang F (2023) Micro- and nanoplastics in soil ecosystems: analytical methods, fate, and effects. TrAC Trends Anal Chem 169:117309. 10.1016/j.trac.2023.117309

[CR63] Woodward EE, Hladik ML, Orlando JL (2021) Insecticide and fungicide concentrations in irrigation runoff and soils from a lettuce field in the Salinas Valley, California, 2019 and 2020. U.S. Geological Survey data release, 10.5066/P9ACMDRK.

[CR64] Xu G, Liu Y, Song X, Li M, Yu Y (2021) Size effects of microplastics on accumulation and elimination of phenanthrene in earthworms. J Hazard Mater 403:123966. 10.1016/j.jhazmat.2020.123966 33265007 10.1016/j.jhazmat.2020.123966

[CR65] Xu T, Dyer DG, McConnell LL, Bondarenko S, Allen R, Heinemann O (2015) Clothianidin in agricultural soils and uptake into corn pollen and canola nectar after multiyear seed treatment applications. Environ Toxicol Chem 35(2):311–321. 10.1002/etc.3281 10.1002/etc.3281 PMC4736462 26467536

[CR66] Xu Z, Qian X, Wang C, Zhang C, Tang T, Zhao X, Li L (2020) Environmentally relevant concentrations of microplastic exhibits negligible impacts on thiacloprid dissipation and enzyme activity in soil. Environ Res 189:109892. 10.1016/j.envres.2020.109892 32678737 10.1016/j.envres.2020.109892

[CR67] Zhang B, Chao J, Chen L, Liu L, Yang X, Wang Q (2021) Research progress of nanoplastics in freshwater. Sci Total Environ 757:143791. 10.1016/j.scitotenv.2020.143791 33280859 10.1016/j.scitotenv.2020.143791

[CR68] Zhou S, Song J, Sun H, Jiang Y, Jia H, Wang J, Yin X (2024) Transport of polyethylene and polypropylene microplastics under the action of agricultural chemicals: role of pesticide adjuvants and neonicotinoid active ingredients. Environ Res 252:118975. 10.1016/j.envres.2024.118975 38649018 10.1016/j.envres.2024.118975

[CR69] Zhu B, Fang Y, Zhu D, Christie P, Ke X, Zhu Y (2018) Exposure to nanoplastics disturbs the gut microbiome in the soil oligochaete *Enchytraeus crypticus*. Environ Pollut 239:408–415. 10.1016/j.envpol.2018.04.017 29679938 10.1016/j.envpol.2018.04.017

[CR70] Zhu D, Chen Q, An X, Yang X, Christie P, Ke X, Wu L, Zhu Y (2017) Exposure of soil collembolans to microplastics perturbs their gut microbiota and alters their isotopic composition. Soil Biol Biochem 116:302–310. 10.1016/j.soilbio.2017.10.027

