# Supplementary material for: Single and combined chronic toxicity of polystyrene nanoplastics (PSNP) and clothianidin on collembolans and enchytraeids
Source: Environ Sci Pollut Res Int. 2026 Mar 3;33(10):4165–74. doi: 10.1007/s11356-026-37540-2 (PMC13053340; doi:10.1007/s11356-026-37540-2)
Supplement: Supplementary file 1 — (DOCX 207 KB) [file 11356_2026_37540_MOESM1_ESM.docx]

**Single and combined chronic toxicity of polystyrene nanoplastics (PSNP) and clothianidin on collembolans and enchytraeids**

Felipe Ogliari Bandeira ^a, b, d^, Paulo Roger Lopes Alves ^b^, Tamires Rodrigues dos Reis ^c^, Dilmar Baretta ^d^, Carolina Riviera Duarte Maluche Baretta ^e^, William Gerson Matias ^a,*^

^a^ Laboratory of Environmental Toxicology, Department of Sanitary and Environmental Engineering, Federal University of Santa Catarina, Florianópolis (SC), 88040-970, Brazil

^b^ Laboratory of Soil Ecotoxicology, Universidade Federal da Fronteira Sul, Chapecó (SC), 89815-899, Brazil

^c^ Department of Soil Science, Federal University of Lavras, Lavras (MG), 37200-900, Brazil

^d^ Santa Catarina State University, Center for Higher Education of the West, Department of Animal Science, 89815-630, Chapecó (SC), Brazil

^e^ Graduate Program in Environmental Sciences, Community University of Chapecó Region –Unochapeco, 89809-900, Chapecó (SC), Brazil

* To whom correspondence should be addressed (william.g.matias@ufsc.br)

Corresponding author: William Gerson Matias.

Laboratory of Environmental Toxicology, Department of Sanitary and Environmental Engineering, Federal University of Santa Catarina, Mailbox 476, CEP 88.010-970, Florianópolis, SC, Brazil.

**Figure S1** Results of the preliminary assay with collembolans *Folsomia candida* and enchytraeids *Enchytraeus crypticus* exposed to Entisol contaminated with increasing concentrations of polystyrene nanoplastic (PSNP) or clothianidin (CLO).

**

**

**Table S1** EC_50_ values obtained from the preliminary tests with collembolans *Folsomia candida* and enchytraeids *Enchytraeus crypticus* exposed to Entisol contaminated with increasing concentrations of polystyrene nanoplastic (PSNP) or clothianidin (CLO). These results were used to define the Toxic Units adopted in the final tests (1 TU = EC_50_).

| Species | EC_50_ (mg kg^-1^) | |
| --- | --- | --- |
|  | PSNP | CLO |
| *E. crypticus* | 256 (209 - 303) | 2.67 (2.37 – 2.97) |
| *F. candida* | 75 (59 – 90) | 0.045 (0.040 – 0.051) |

**Table S2** Soil moisture and pH from the beginning (day 0) and from the end of the chronic toxicity assays with *Enchytraeus crypticus* and *Folsomia candida* exposed to polystyrene nanoplastic (PSNP), clothianidin (CLO), and their mixtures in Entisol.

| Species - test duration | PSNP  (mg kg^-1^) | CLO  (mg kg^-1^) | Initial pH | Final  pH | Initial soil moisture  (% WHC) | Final soil moisture  (% WHC) |
| --- | --- | --- | --- | --- | --- | --- |
| *E. crypticus* - 21d | 0 | - | 4.28 | 4.25 | 55.0 | 55.3 |
|  | 64 | - | 4.26 | 4.25 | 57.1 | 55.4 |
|  | 128 | - | 4.24 | 4.23 | 56.4 | 53.8 |
|  | 192 | - | 4.20 | 4.26 | 57.1 | 56.3 |
|  | 256 | - | 4.24 | 4.24 | 56.5 | 54.5 |
|  | 384 | - | 4.18 | 4.25 | 56.7 | 54.8 |
|  | 512 | - | 4.16 | 4.22 | 55.8 | 57.4 |
|  | - | 0 | 4.28 | 4.15 | 55.0 | 55.3 |
|  | - | 0.67 | 4.28 | 4.15 | 57.5 | 60.4 |
|  | - | 1.33 | 4.25 | 4.20 | 57.4 | 53.7 |
|  | - | 2.00 | 4.20 | 4.16 | 57.4 | 57.9 |
|  | - | 2.67 | 4.18 | 4.13 | 57.4 | 55.6 |
|  | - | 4.00 | 4.15 | 4.13 | 57.2 | 56.2 |
|  | - | 5.34 | 4.14 | 4.13 | 57.2 | 56.9 |
|  | 0 | 0 | 4.28 | 4.25 | 55.0 | 55.3 |
|  | 64 | 0.67 | 4.08 | 4.42 | 58.2 | 55.1 |
|  | 128 | 1.33 | 4.52 | 4.32 | 56.4 | 55.4 |
|  | 192 | 2.00 | 4.45 | 4.37 | 56.1 | 57.9 |
|  | 256 | 2.67 | 4.41 | 4.32 | 56.3 | 61.4 |
|  | 384 | 4.00 | 4.39 | 4.37 | 56.7 | 55.1 |
|  | 512 | 5.34 | 4.34 | 4.36 | 56.2 | 58.2 |
| *F. candida* - 28d | 0 | - | 4.84 | 4.25 | 59.3 | 58.5 |
|  | 18.75 | - | 4.59 | 4.27 | 57.2 | 57.7 |
|  | 37.50 | - | 4.30 | 4.50 | 47.6 | 58.8 |
|  | 56.25 | - | 4.27 | 4.44 | 57.3 | 59.0 |
|  | 75.00 | - | 4.29 | 4.42 | 59.8 | 58.9 |
|  | 112.50 | - | 4.27 | 4.32 | 56.9 | 58.6 |
|  | 150.00 | - | 4.30 | 4.34 | 60.4 | 58.7 |
|  | - | 0 | 4.84 | 4.25 | 59.3 | 58.5 |
|  | - | 0.01 | 4.38 | 4.97 | 58.2 | 58.3 |
|  | - | 0.02 | 4.64 | 4.86 | 58.7 | 57.7 |
|  | - | 0.03 | 4.28 | 4.73 | 73.2 | 58.0 |
|  | - | 0.04 | 4.29 | 4.68 | 46.5 | 57.3 |
|  | - | 0.07 | 4.61 | 4.63 | 59.4 | 57.3 |
|  | - | 0.09 | 4.13 | 4.63 | 59.2 | 58.3 |
|  | 0 | 0 | 4.84 | 4.25 | 59.3 | 58.5 |
|  | 18.75 | 0.01 | 4.72 | 4.60 | 56.4 | 58.7 |
|  | 37.50 | 0.02 | 4.50 | 4.52 | 58.5 | 58.0 |
|  | 56.25 | 0.03 | 4.90 | 4.39 | 57.6 | 64.0 |
|  | 75.00 | 0.04 | 4.33 | 4.52 | 57.3 | 58.1 |
|  | 112.5 | 0.07 | 4.21 | 4.43 | 59.2 | 56.4 |
|  | 150.00 | 0.09 | 4.14 | 4.42 | 57.5 | 57.1 |

WHC - Water holding capacity.
